# Pathway to care for drug resistant tuberculosis cases identified during a retrospective study conducted in high TB burden wards in Mumbai

**DOI:** 10.12688/gatesopenres.12785.2

**Published:** 2018-05-10

**Authors:** Eunice Lobo, Shimoni Shah, Sheela Rangan, Yatin Dholakia, Nerges Mistry

**Affiliations:** 1 The Foundation for Medical Research, Mumbai, 400018, India

**Keywords:** Drug resistant Tuberculosis, Mumbai, delays, pathway to TB care

## Abstract

**Background:** Mumbai is witnessing a rising incidence of all forms of drug resistant tuberculosis (DR-TB).

**Methods:** A population-based, retrospective study was conducted between April and July 2014, in 15 high TB burden wards in Mumbai, to capture the patient pathways to TB care. A total of 23 DR-TB patients were identified and their pathways to access DR-TB care were recorded using semi-structured interviews.

**Results:** The total DR-TB pathway time of new patients (who did not report any past episode of TB) (180 days; IQR 123,346) was found to be more than twice that of retreatment patients (who reported a past episode of TB) (69 days; IQR 42,128).

**Conclusions:** The unacceptable delay for diagnosis and treatment of DR-TB in Mumbai advocates for consistent implementation of early screening of patients using rapid gene-based technologies.

## Introduction

The rising threat of multidrug resistant-tuberculosis (MDR-TB), defined as *in vitro* resistance to at least isoniazid and rifampicin, necessitates early detection of drug resistance and appropriate treatment initiation. A total of 480,000 MDR-TB cases are identified annually worldwide, accounting for 3.3% of newly diagnosed TB patients and 20% of retreatment TB patients ^1, 2^. An estimated 71,000 MDR-TB cases are reported from India, making it not only a major public health threat, but a huge economic burden on patients and health-systems as well. Mumbai, a fast growing urban metropolis in India with about 60% population living in vulnerable settings, has become the epicenter of various forms of MDR-TB ^3^. Whilst national estimates for MDR-TB are 2.5% among new and 16% among retreatment patients ^1^, reports from the city have recorded rates as high as 24% among new and 41% in retreatment patients ^4^.

A patient with TB continues to be infectious until initiated on effective treatment. It is therefore imperative to understand the amount of time taken to detect patients with DR-TB and initiate them on appropriate treatment. This study looks at the durations from the onset of symptoms until initiation of appropriate treatment and tries to understand the type of patients that show maximum delay in accessing care.

## Methods

### Study design and participants

Between April and July 2014, a population-based, two-stage, retrospective study was conducted in 15 high TB burden wards. BMGF consultants had provided the estimated sample size of around 100 TB cases based on TB prevalence surveys conducted in rural areas (N=D*Z2(p*q)/(e2)).

The first stage involved identification of patients treated for TB with a household (HH) survey, using a multistage cluster approach from the 2011 Census Enumerated Block (CEB) maps. Fifteen wards falling under MCGM consisting of both slum and non-slum areas were identified. Census Enumeration Blocks (CEB) maps were used as the reference point for the primary sampling unit (PSU) in the urban area. The CEB map consisted of 120–180 HHs in each block and helped demarcate the slum areas and the non-slum areas. The CEB in these selected 15 wards acted as the sampling frame for the survey. Assuming an average cluster size of 160 per CEB, 100 Urban Frame Survey (UFS) blocks were selected for the primary objective of conducting a survey of over 10,000 HHs. Going by the assumption that the CEB blocks have a clear demarcation of slum and non-slum areas, sampling frames consisting of the slum based CEBs were created. The blocks from each ward were selected proportionate to the total number of HHs in the slums of the 15 wards. The required number of PSUs (100 CEBs) was selected randomly from the list of all slum based CEBs for each of the wards. In a selected CEB, all HHs in the selected block unit were enumerated.

Cases of TB were identified by means of two questions. One that recorded details of all the cases of cough in the family and narrowed down to asking the family member if the doctor they had consulted had told them that they had TB on the basis of tests conducted. The other question that captured the TB cases pertained to those that had occurred in the past six months who due to some treatment being taken did not show any signs of coughing any more. Around 21,016 HHs were listed, of which 14,250 (68%) agreed for an interview. From these participating HHs, a total of 153 TB cases were drawn.

The second stage involved in-depth interviews of identified TB patients who were treated for pulmonary TB in Mumbai and had completed their anti-TB treatment in the past six months. A total of 82 patients consented to being interviewed using a pre-tested open-ended semi-structured interview schedule ( Supplementary File 1). Pre-testing was conducted as per study protocol on six known TB cases from K/East ward who were excluded from the final study sample. Of the 82 patients that consented to be interviewed, 23 DR-TB patients were identified (28%), and only these interviews were included in the present analysis. The data from the remaining 59 patients has been previously published ^5^. Patients were identified as DR-TB cases if they had completed their anti-DR-TB treatment in Mumbai within the past six months of the interview. Besides patient information, diagnosis and treatment records of patients were obtained and seen by the researchers. Photographs of these were taken and shown to our clinical consultant on the study (YD), on whose opinion, the cases were classified as DR-TB. Two were identified as extensively drug resistant (XDR) cases based on their line probe assay (LPA) (Hain Lifescience, Nehren-Germany) results. Monoresistance to Isoniazid (INH) could not be identified as drug sensitivity testing (DST) through the line probe assay LPA was not available for all cases at the time of the study.

**Figure 1.**
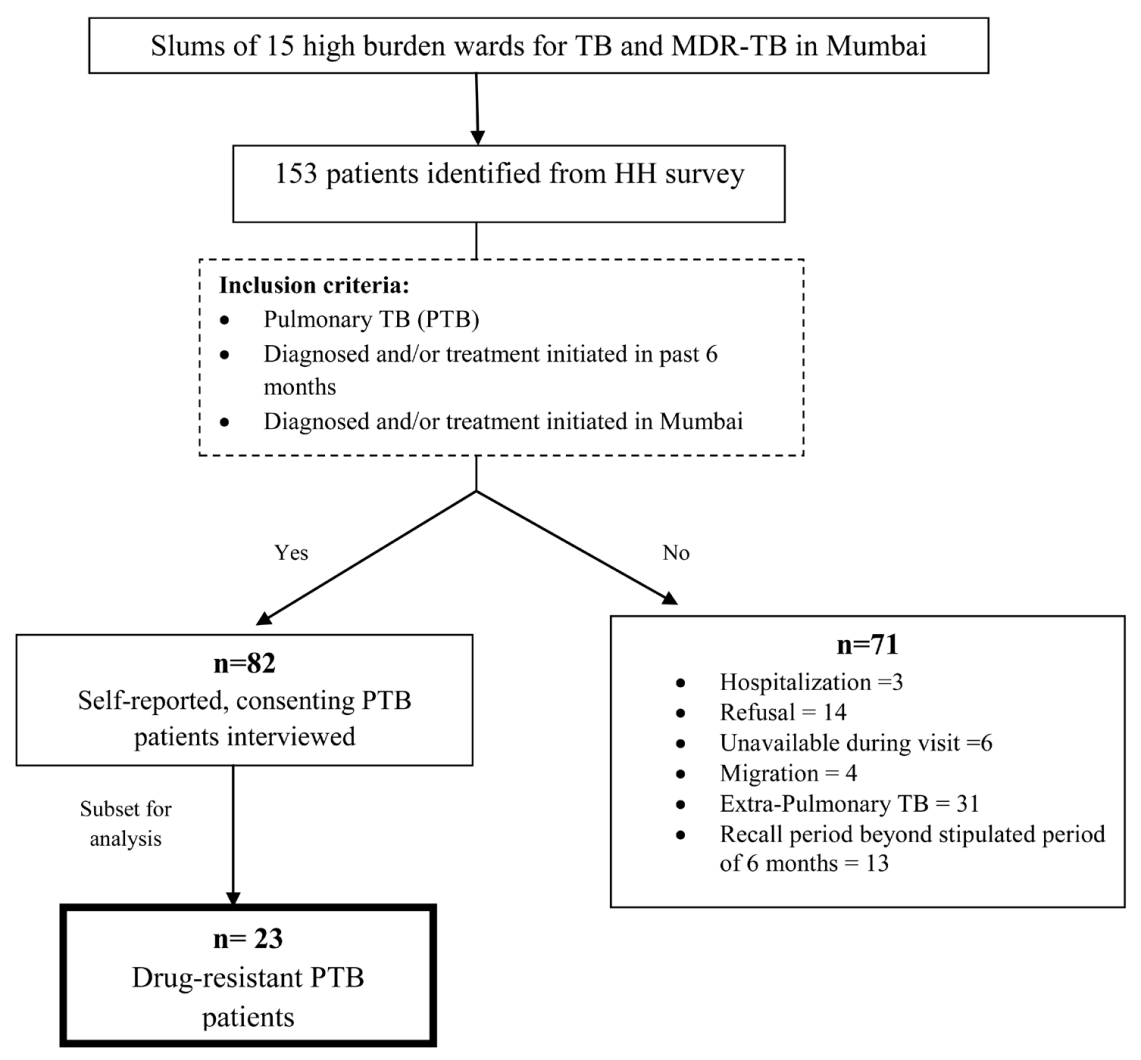
Patients selected for subset analysis of DR-TB pathway to care. The figure depicts the selection of patients for inclusion in the study and subset analysis presented.

Figure 1 shows the selection flowchart for the participants.

The 23 patients that were included in this study came from 10 of the 15 high burden TB wards namely: M/East (8 patients), H/East (2 patients), M/West (2 patients), F/North (2 patients), P/North (1 patient), G/North (2 patients), R/South (1 patient), L (1 patient), N (3 patients), S (1 patient).

All patient interviews were conducted at the participants’ residence by trained health researchers.

### Data collection

Patients were interviewed using a semi structured interview guide ( Supplementary File 1) in their preferred local language (Hindi or Marathi) at a time and date convenient to them. Patient anonymity regarding name and address was maintained through a unique identification number. Interviews were audio recorded.

At the end of the interview, quantitative data were filled on physical formats by the researchers ( Supplementary File 2). For the purpose of quality check, three levels of verification by listening to recorded interviews were conducted. First, each researcher team cross-checked the quantitative data forms of another research team. Further 25% interviews were cross-checked by senior researchers for errors, and finally a set of random 10% interviews were checked by consultants to the study.

### Statistical analysis

The data was entered in CSPro v5, and exported into SPSS v19 (SPSS, Inc., Chicago, IL, USA) for analysis. Although other data was collected, it was not assessed herein as the purpose of this publication was to focus solely on the durations and testing practices followed. The total time taken from onset of TB symptoms to first care-seeking and until initiation of DR-TB treatment was estimated by dates collected for various events and presented as medians, means and interquartile range (in days). Median differences in pathways for new and re-treatment patients were compared using Mann Whitney U-test with significance established at P values ≤ 0.05.

### Ethical statement

Ethical approval was obtained from the Institutional Ethics Committee (IEC) of the Foundation for Medical Research (vide IEC no. FMR/IEC/TB/01/2013).

Verbal informed consent for answering the survey was obtained from individuals who underwent the HH survey in stage 1 sampling. Following the verbal consent, field researchers then contacted the patients over the phone to schedule in-depth interviews. On meeting the patients, written informed consent was first obtained for the in depth interviews after which 82 patients who consented were included in the semi-structured interview. In the case of minors, written consent was obtained from their care-givers (parents/guardians). The consent obtained included participation in the interview, digital audio recording and note keeping, reviewing of patient’s TB treatment-related documents and permission to publish anonymised data in any report, journal, etc.

## Results

The 23 DR-TB patients included 14 males and 9 females. Mean age group of the patients was 29 years with a majority of the patients (n=17) aged between 16–34 years, 4 patients between 35–54 years and 1 patient aged 14 years and 65 years each. Only 5 patients admitted to having addictions like tobacco, alcohol, recreational drugs and three reported having chronic co-morbid conditions with two being HIV positive and one asthmatic.

Thirteen of the 23 patients interviewed (56%) were retreatment patients, of whom only four (31%) were advised DST upon their initial presentation with TB symptoms. Nine (69%) of the retreatment patients were initiated on first line treatment before being diagnosed and treated as DR-TB. Of the 10 new patients with no past history of TB, only two were advised DST upon initial presentation with TB symptoms and the remaining eight (80%) were treated with first line anti TB medicines. Preference for Mumbai’s strong and robust public sector for TB treatment was seen among the interviewed patients, with a significant shift from first seeking care from the private sector (n=19, 83%) for initial symptoms, to approaching the public sector (n=16, 70%) for diagnosis and treatment of DR-TB.

Only four patients (17%) were diagnosed with DR-TB using molecular tests like GeneXpert and LPA, facilities for which were available in both, the public and private sectors. Sixteen patients (70%) were diagnosed using only culture based / phenotypic tests. A combination of molecular and phenotypic tests were used for diagnosis in only two patients (9%) and the remaining three (13%) were presumptively diagnosed using only chest x-ray and sputum examination for acid fast bacilli. Due to lack of phenotypic testing facilities in the public sector, where a majority of the patients were diagnosed, it is most likely that the samples were sent to private labs for testing.

**Figure 2.**
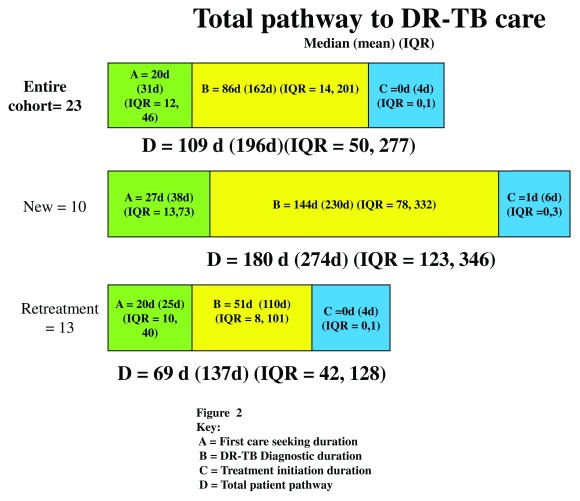
Total pathway to DR-TB care of the entire cohort. The figure depicts the pathway to DR-TB care for patients from the entire cohort, along with new patients and retreatment patients.

Figure 2 depicts the median (mean) durations and interquartile range in days for time taken from onset of symptom to first point of care, first point of care to DR-TB diagnosis and from DR-TB diagnosis to initiation of DR-TB treatment, for the entire cohort and for new and retreatment patients.

**Figure 3.**
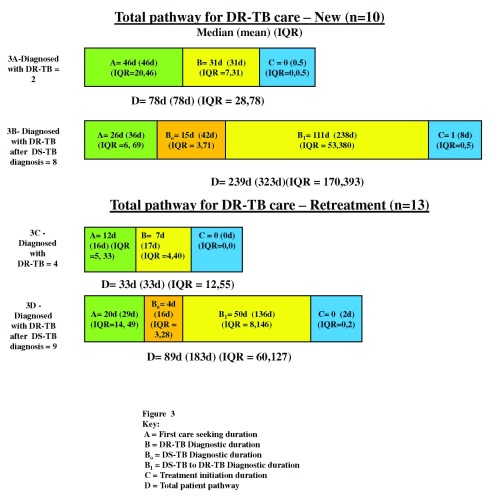
Total pathway to DR-TB care of new and retreatment patients. The figure depicts the pathway to DR-TB care for new and retreatment patients for patients diagnosed with DR-TB at presentation (A and C) and patients diagnosed after a course of first line treatment (B and D).

After the patients first sought care, the average time taken to diagnose and initiate DR-TB treatment was 87 days (IQR 17-202) for the entire cohort (data not shown in figure). Further analysis was undertaken to see the median difference in pathways of new and retreatment DR-TB patients ( Figure 2). The time taken from first care seeking to DR-TB diagnosis (p value = 0.041) and the total pathway duration (p value = 0.016) were significantly shorter for retreatment patients. However the duration from onset of symptoms to first care seeking was almost similar for the two groups, indicating that patients with a past episode of TB were not seeking care earlier compared to new patients. Patients were further split into those diagnosed with DR-TB at presentation and those diagnosed after a course of first line treatment ( Figure 3).

While no significant differences in pathway durations were observed after splitting the patients, (due to the small number of patients in each group), new patients initially diagnosed and treated as drug susceptible patients showed the longest median pathway of eight months ( Figure 3B). The study was not able to assess if the patients progressed from drug sensitive tuberculosis (DS-TB) to DR-TB or were incorrectly diagnosed with DS-TB. The shortest median pathway of one month was seen in retreatment patients who were diagnosed with DR-TB initially ( Figure 3C).

## Discussion

With respect to the entire cohort the median pathways after seeking first access to care was 86 days (IQR 14-202) which is relatively shorter than that reported in a multistate study (128 days, IQR 103-173) ^6^ but nevertheless long. Even though the study findings show certain significant differences in pathway durations of new and re-treatment DR-TB patients, we caution that in light of the small sample size, the observations currently be interpreted as indicative. The study throws light on patient related delay, seen specifically among retreatment patients who showed a similar time frame in accessing first care (20 days vs 27 days) on developing symptoms that were probably similar to their first disease episode. This could be due to patient denial of the disease and for lack of information/counseling received from their TB care providers in the past ^5^.

Since it is more likely for a retreatment patient to be resistant at their second episode, DST testing is mandated at the time of diagnosis. However, the failure in undertaking this in over 70% of patients in the present study, exceeded the proportion (45%) reported in another study conducted in Andhra Pradesh, India ^7^. This calls for a more stringent implementation of the diagnostic algorithms for standards of care in DR-TB at field level. This is particularly important for areas with high prevalence of DR-TB such as Mumbai.

The unacceptably long pathways for diagnosis and treatment of DR-TB in Mumbai advocates for stronger implementation of early screening of patients for DR-TB through use of rapid gene-based technologies. Patients with the least duration were the ones laboratory diagnosed with DR-TB prior to initiation of therapy. This advocates for the use of DR testing at the time of presentation of the patients to reduce their total pathway. This seems to be well on track with the number of GeneXpert machines available in the city increasing from eight when the study was initiated in 2015, to 19 so far in 2017. There is also a new policy dated 1 ^st^ Jan, 2018 which mandates all new cases to be tested with GeneXpert at time of presentation. Focus needs to be on people residing in vulnerable settings, contacts of DR-TB cases, immune-compromised patients and those living in compromised housing. Contemporary technologies need to be rapidly made available in the public sector and extended to patients seeking care from the private sector as well.

## Data availability

Raw data in Excel format of the 23 drug resistant TB cases identified in Mumbai and the semi structured interview guide that was used to collect the data from the identified DR-TB patients are available on OSF: http://doi.org/10.17605/OSF.IO/8CTBV ^8^

Data are available under the terms of the Creative Commons Zero "No rights reserved" data waiver (CC0 1.0 Public domain dedication).

## Supplementary material

Supplementary File 1: Semi structured interview schedule.

Supplementary File 2: Quantitative data sheet.
