# Supplementary material for: Pathway to care for drug resistant tuberculosis cases identified during a retrospective study conducted in high TB burden wards in Mumbai
Source: Gates Open Res. 2018 May 10;2:9. Originally published 2018 Feb 19. [Version 2] doi: 10.12688/gatesopenres.12785.2 (PMC5974602; doi:10.12688/gatesopenres.12785.2)
Supplement: Supplementary file 1 [file gatesopenres-2-13891-s0000.tgz › a7c2753c-20b5-439f-80dc-96dafec7ff07.docx]

**PATIENT PATHWAY TO TUBERCULOSIS CARE IN MUMBAI & PATNA**

In-depth interview questionnaire for known TB cases

1. **Section One : Patient profile**

Patient and his/her family details, also include consumption of any additive substance, comorbidities.

Amenities present in the house (partly observation-based, if conducted at interviewee’s house)

1. **Section Two : Patient health seeking behaviour for the current episode of illness ( Tuberculosis )**

First point of care sought for any illness among any member of the family

Basic TB awareness

Development of symptoms – **Date of First onset of symptoms** and other symptoms prior to being diagnosed

Actions taken after symptom(s) developed

Reason for seeking medical care at that point in time

1. **Section Three : Pathway to care for the current episode of illness**

**Individual provider details to be asked**

**Date of approaching first provider/ facility and his/ her / facility details** (refer to provider code)

**Type of tests recommended by the provider (names),**

**Number of days elapsed between the first visit to the provider and the ordering of tests,** Continued with provider after recommending the tests/ conducting the tests- if no, **date of leaving the provider (**reason for leaving the provider**)**

**What medication/ treatment was prescribed before ordering the tests- duration of medication/treatment, type of medications prescribed and number of visits made to the provider during this time**

**Were the test results collected, if yes did the patient visit the provider with the results and after how many days? Reason for not approaching immediately with the test results. Did the patient continue visiting the same provider, if no what was the date of leaving the provider?**

**After how many days was the next provider approached**

**Duration of time taken by the provider after the initial examination to tell the patient what he was suffering from?** Was the same provider continued? If yes, **duration of time taken to start the treatment after the diagnosis.** If no, **date of leaving the provider (**reason for leaving the provider**)**

**How long was the given treatment regimen and how long did the patient continue it? Was MDR-TB suspected? What drugs were prescribed (no. of drugs and injections taken)? Total number of visits made to the provider? Was the patient hospitalised. If yes, what was the duration?** Was there any improvement in patient’s health? If no, was the same provider continued? **Date of leaving provider (**reason for leaving the provider**)**

Advice and counselling related to TB provided by the above provider, any tests recommended for other illnesses/conditions like HIV/Diabetes and / or follow up sputum tests, any advice related to children below 6 years in the HH

Overall experience with the provider

3 things liked and disliked about the provider

1. **Section Four : Costs incurred while seeking care for TB**

Follow the questionnaire and enter details wherever applicable

**How much did the patient spend on each provider before being diagnosed with TB, including the visit when the patient actually received the TB diagnosis?** (Enter the details in the given table)

Guardian/accompanying person costs

Hospitalization costs

Other costs- Food supplements

Other costs – Illnesses

Insurance

Coping costs

Socio-economic information about individuals and change in income or lost productivity due to TB

House hold income and spending

Tangible costs i.e. did the illness affect the patient’s social or private life?

1. **Section Five : Checklist to be filled at the end of the interview**

For each individual provider photograph the following:

Any notes written by the provider

Lab results or imaging results from the provider

Prescription given by the provider

TB medication blister packs

Others (specify): _____________
