# Supplementary material for: Pathway to care for drug resistant tuberculosis cases identified during a retrospective study conducted in high TB burden wards in Mumbai
Source: Gates Open Res. 2018 May 10;2:9. Originally published 2018 Feb 19. [Version 2] doi: 10.12688/gatesopenres.12785.2 (PMC5974602; doi:10.12688/gatesopenres.12785.2)
Supplement: Peer review history

# Peer review: Referee response for version 2

**DOI:** 10.21956/gatesopenres.13891.r26435

**Published:** 2018-05-29

**Authors:** Amrita Daftary

**Affiliations:** 1 McGill International TB Centre, McGill University, Montreal, QC, Canada

The authors have adequately responded to the reviewer comments.

I have read this submission. I believe that I have an appropriate level of expertise to confirm that it is of an acceptable scientific standard.

# Peer review: Referee response for version 2

**DOI:** 10.21956/gatesopenres.13891.r26434

**Published:** 2018-05-11

**Authors:** Ramnath Subbaraman

**Affiliations:** 1 Department of Public Health and Community Medicine, Tufts University School of Medicine, Boston, MA, USA

The authors have done an excellent job addressing all the concerns raised in my initial review.

I have read this submission. I believe that I have an appropriate level of expertise to confirm that it is of an acceptable scientific standard.

# Peer review: Referee response for version 1

**DOI:** 10.21956/gatesopenres.13845.r26256

**Published:** 2018-03-26

**Authors:** Ramnath Subbaraman

**Affiliations:** 1 Department of Public Health and Community Medicine, Tufts University School of Medicine, Boston, MA, USA

This is an important study that is a unique contribution for a few reasons. First, while numerous studies have evaluated delays in care-seeking for TB patients, fewer studies have looked at this issue in drug-resistant TB patients. Second, very few studies have used a population-based sampling approach to identify patients with TB and then to evaluate delays, since it is much easier to identify TB patients for these studies after they have been diagnosed at health facilities. While costly and time-intensive, using a population-based sampling approach may reduce biases that occur when recruiting patients who get diagnosed at particular health facilities. Third, the scale of this study is also unique—covering 15 city wards in Mumbai. While the sample size is relatively small for this study, this is understandable given the considerable challenges of identifying drug-resistant TB patients using a population-based approach.

 The key finding of this paper - the considerable difference in delay between drug-resistant TB patients who were “new” versus “retreatment” cases is an important one. The figures, while a bit challenging to interpret initially, really bring home the authors’ points regarding differences in delays between groups of patients.

 However, the following aspects of the manuscript could be improved: 

1. **Details of the population-based sampling approach:** Very few details (<1 paragraph) are provided regarding the population-based sampling approach used to identify drug-resistant TB patients on treatment in the community. One of the criteria for evaluation for this journal is that the manuscript provides “sufficient details to allow replication.” For others to replicate this work, it would be helpful for the authors to provide more details on: (a) how randomized sampling of clusters within census blocks was conducted; (b) how many clusters the survey was conducted in and rough estimate of cluster sizes in number of households; and (c) how screening of household members was conducted to identify patients on TB therapy - including examples of the questions asked to identify patients and whether each individual in the household was asked questions directly or whether only the head of household was asked for all members.
2. **Reasons for low response rate and comparison of demographics:** If the authors have a sense of why the consent rate for the study was relatively low for identified TB patients (82 out of 153) it would be helpful to provide some information on this, even if not collected formally through a survey. Also, if any demographic details were collected on patients who did not consent, a comparison of these details for patients who consented versus those who didn’t would be helpful to see if those who participated were similar to the larger population of patients identified.
3. **Inclusion of a clear case definition:** It would be helpful to include a brief (2 to 3 sentence) case definition in the methods section clarifying exactly how you classified patients as being DR-TB patients. From the results section, it appears that 20 out of 23 patients had DR-TB based on laboratory confirmation (either culture, CB-NAAT, or LPA) but the remaining 3 were diagnosed with DR TB empirically. If so, how did the interviewers and researchers determine and confirm that these patients were being treated as DR-TB cases? Based on self-report? Or based on their medication regimen? It would be helpful to clarify this case definition and how you decided 23 out of 82 patients were DR-TB patients.
  Also, for patients with DR-TB based on culture results, did you only include MDR TB patients in this sample of 23 patients (i.e., resistant to INH and rifampicin at minimum) or did you also include patients with INH monoresistance? I am presuming that you are really only referring to MDR TB patients based on culture and rifampin-resistant TB patients based on CB-NAAT, but please clarify.
4. **Test for statistical significance:** The difference between the median and mean in most of the samples and sub-samples suggest that the values for delays might not follow a normal distribution. This is a relatively minor point, but I wonder whether it might be more appropriate to use a Wilcoxon-Mann-Whitney test rather than a t test as the test of significance for the various analyses.
5. **The first paragraph of the results section is confusing: **When you say that only 4 of 13 retreatment patients underwent drug susceptibility testing and only 2 of 10 new patients underwent drug susceptibility testing. But clearly 20 of 23 patients ultimately underwent drug susceptibility testing later in their clinical course to get diagnosed with DR TB based on what you say later. As such, I think what you mean to say in that first paragraph is that only 4 of 13 retreatment and 2 of 10 new patients underwent drug susceptibility testing upon their initial presentation with TB symptoms.
6. **The discussion section does not extrapolate on the key finding of the considerable difference in delays between new and retreatment patients:** This is the main finding highlighted in your abstract and in Figure 2; however, you do not provide more insight into this finding in the Discussion section. I think that this finding really highlights the terrible delays faced by DR TB patients without a prior TB history, and it may suggest that even new TB patients should be screened upfront with tests that include drug susceptibility testing, at least in the Mumbai setting.
7. Inclusion of a summary of the demographic details of the participants included in the study - that is, breakdown by age, gender, socioeconomic status in a small table or paragraph - would be helpful.

I have read this submission. I believe that I have an appropriate level of expertise to confirm that it is of an acceptable scientific standard, however I have significant reservations, as outlined above.

# Response

**Published:** 2018-04-26

**Authors:** Nerges Mistry

**Affiliations:** The Foundation for Medical Research, India

**1. Response:** Thank you for your comment. We have included further details on the HH cluster sampling and questions used to screen the patients. This has been added in the Methods section on page 3 in track change mode. It reads as, ***‘*** ***Fifteen wards*** ***falling under MCGM consisting of both slum and non-slum areas were identified. Census Enumeration Blocks (CEB) maps were used as the reference point for the primary sampling unit (PSU) in the urban area. The CEB map consisted of 120-180 HHs in each block and helped demarcate the slum areas and the non-slum areas. The CEB in these selected 15 wards acted as the sampling frame for the survey. Assuming an average cluster size of 160 per CEB, 100 Urban Frame Survey (UFS) blocks were selected for the primary objective of conducting a survey of over 10,000 HHs. Going by the assumption that the CEB blocks have a clear demarcation of slum and non-slum areas, sampling frames consisting of the slum based CEBs were created. The blocks from each ward were selected proportionate to the total number of HHs in the slums of the 15 wards. The required number of PSUs (100 CEBs) was selected randomly from the list of all slum based CEBs for each of the wards. In a selected CEB, all HHs in the selected block unit were enumerated.***

***TB cases were identified by means of two questions. One that recorded details of all the cases of cough in the family and narrowed down to asking the family member if the doctor they had consulted had told them that they had TB on the basis of tests conducted. The other question that captured the TB cases pertained to those that had occurred in the past six months who due to some treatment being taken and did not show any signs of coughing any more. Around 21,016 HHs were listed, of which 14,250 (68%) agreed for an interview. From these participating HHs, a total of 153 TB cases were drawn.’***

**2. Response:** We have modified Figure 1. to give the number of cases excluded for various reasons. Only 14 did not consent to participate (refusals) whereas others either did not meet the inclusion criteria or were unable to be interviewed. The inclusion criteria for the second stage was more stringent as opposed to a more broader criteria used in selection at stage 1. Unfortunately demographic details for patients that did not consent or not included in the study were not collected as it did not fall in the purview of the study objective.

**3. Response:** Thank you for your comment. We have clarified this in the methods section, page 4 which reads as, ***‘*** ***Patients were identified as DR-TB cases if they had completed their anti-DR-TB treatment in Mumbai within the past six months of the interview. Besides patient information, diagnosis and treatment records of patients were obtained and seen by the researchers. Photographs of these were taken and shown to our clinical consultant on the study (YD), on whose opinion, the cases were classified as DR-TB. Two cases were identified as XDR cases based on their LPA/DST results. Monoresistance to INH could not be identified as LPA was not available for all cases at the time of the study.’***

**4 . Response:** Thank you for bringing this to our notice. We had analyzed the median differences based on Mann-Whitney U test as mentioned in the results section. However, due to over-sight this was mis-represented in the methods section as t-test. Change in statistical test is reflected in the methods section under statistical analysis sub-section.

**5. Response:** Thank you for your comment. As you rightly mentioned, we do mean that the patients underwent DST upon their initial presentation with TB symptoms. We have modified the text in the results section, page 7 (paragraph 2) which now reads as, ***‘Thirteen of the 23 patients interviewed (56%) were retreatment patients, of whom only four (31%) were advised drug sensitivity testing (DST) upon their initial presentation with TB symptoms.’***  And ***‘Of the 10 new patients with no past history of TB, only two were advised DST upon initial presentation with TB symptoms.’***

**6. Response: **Thank you. Based on your suggestion, we have included additional details in the discussion which now reads as,

***‘With respect to the entire cohort the median pathways after seeking first access to care was 86 days (IQR 14-202) which is relatively shorter than that reported in a multistate study (128 days, IQR 103-173)*** ***^6^*** ***but nevertheless long. Even though the study findings show certain significant differences in pathway durations of new and re-treatment DR-TB patients, we caution that in light of the small sample size, the observations currently be interpreted as indicative. The study throws light on patient related delay, seen specifically among retreatment patients who showed a similar time frame in accessing first care (20 days vs 27 days) on developing symptoms that were probably similar to their first disease episode. This could be due to patient denial of the disease and for lack of information/counseling received from their TB care providers in the past*** ***^5^*** ***.***

***Since it is more likely for a retreatment patient to be resistant at their second episode, DST testing is mandated at the time of diagnosis. However, the failure in undertaking this in over 70% of patients in the present study, exceeded the proportion (45%) reported in another study conducted in Andhra Pradesh, India*** ***^7^*** ***. This calls for a more stringent implementation of the*** ***diagnostic algorithms for standards of care*** ***in DR-TB at field level. This is particularly important for areas with high prevalence of DR-TB such as Mumbai.***

***The unacceptably long pathways for diagnosis and treatment of DR-TB in Mumbai advocates for stronger implementation of early screening of patients for DR-TB through use of rapid gene-based technologies. Patients with the least duration were the ones laboratory diagnosed with DR-TB prior to initiation of therapy. This advocates for the use of DR testing at the time of presentation of the patients to reduce their total pathway. This seems to be well on track with the number of GeneXpert machines available in the city increasing from eight when the study was initiated in 2015, to 19 so far in 2017. There is also*** ***a new policy*** ***dated 1 ^st^ Jan, 2018 which mandates all new cases to be tested with GeneXpert at time of presentation. Focus needs to be on people residing in vulnerable settings, contacts of DR-TB cases, immune-compromised patients and those living in compromised housing. Contemporary technologies need to be rapidly made available in the public sector and extended to patients seeking care from the private sector as well.’***

 The hyperlink to the diagnostic algorithms in standards of TB care has been inserted and not been mentioned under references as per journal norms.

**7. Response**: Thank you for your suggestion. We have added a paragraph on demographic profile of the patients as paragraph 1. It reads as, ***‘The 23 DR-TB patients included 14 males and 9 females. Mean age group of the patients was 29 years with a majority of the patients (n=17) aged between 16-34 years, 4 patients between 35-54 years and 1 patient aged 14 years and 65 years each. Only 5*** ***patients admitted to having addictions like tobacco, alcohol, recreational drugs and three reported having chronic co-morbid conditions with two being HIV and one asthmatic.’***

 Thank you once again for your valuable feedback. We hope to have addressed all your concerns.

 Sincerely,

 Nerges Mistry and Team

# Peer review: Referee response for version 1

**DOI:** 10.21956/gatesopenres.13845.r26258

**Published:** 2018-03-02

**Authors:** Amrita Daftary

**Affiliations:** 1 McGill International TB Centre, McGill University, Montreal, QC, Canada

This is a well-written, timely and relevant article that suggests there is a significant the difference in TB diagnostic delay between primary DRTB cases and retreatment cases. This is a novel findings and the TB scientific community in India would benefit from this knowledge, as well as a a fuller understanding of the reasons underlying these differential delays. I would request a few points of clarification from the authors.

1. It looks like interviews were conducted with a qualitative stance but analyzed quantitatively (open ended responses appear to have been quantified). Some transparency around this would be appreciated because the methods section suggests the interviews were much richer than the data presented.
2. Far more qualitative and categorical data appear to have been collected (as suggested by both supplemental files) but only a few aspects appear to be presented in this article. Similarly, there is no qualitative (or for that matter, objective) data around reasons for delay, or triangulation b/w the quant and qual findings - one would expect this in a typical mixed methods analysis. Perhaps these will be published later. If so, the authors may want to reconsider how to describe the methods and findings so it is clear at the outset what this paper specifically seeks to address. For example, the may want to highlight that although other data were collected, these data will not be assessed herein.
3. This foundational paper ^1^ should be cited and compared against when discussing the current study findings.
4. Were there any significant associations or trends in the data with regards to patients' sociodemographic (especially, gender and income) or other characteristics collected as part of the standardized survey? While time delay is the primary focus of this paper, it appears, it would be helpful to comment if other factors played a role.
5. The abstract suggests a 180 day delay in DRTB diagnosis (1st episode) whereas the results indicate a median 87 day delay (data not shown). Later in the discussion, the number 86 appears. I would advise maintaining some consistency in the ways in which these findings are reported.
6. With a sample size of 23, the authors may want to acknowledge and address the implications of conveying statistically significant results from their small sample.

Thank you.

I have read this submission. I believe that I have an appropriate level of expertise to confirm that it is of an acceptable scientific standard, however I have significant reservations, as outlined above.

## References

[1] SreeramareddyCTQinZZSatyanarayanaSSubbaramanRPaiM: Delays in diagnosis and treatment of pulmonary tuberculosis in India: a systematic review. *Int J Tuberc Lung Dis*.2014;18(3) : 10.5588/ijtld.13.0585 255-266 10.5588/ijtld.13.0585 24670558PMC4070850

# Response

**Published:** 2018-03-19

**Authors:** Nerges Mistry

**Affiliations:** The Foundation for Medical Research, India

**Referee Report** 02 Mar 2018

**Amrita Daftary**, McGill International TB Centre, McGill University, Montreal, QC, Canada 

**Approved with Reservations**

 This is a well-written, timely and relevant article that suggests there is a significant the difference in TB diagnostic delay between primary DRTB cases and retreatment cases. This is a novel findings and the TB scientific community in India would benefit from this knowledge, as well as a a fuller understanding of the reasons underlying these differential delays. I would request a few points of clarification from the authors.

- It looks like interviews were conducted with a qualitative stance but analyzed quantitatively (open ended responses appear to have been quantified). Some transparency around this would be appreciated because the methods section suggests the interviews were much richer than the data presented.

**Response**: Thank you for your comment. As mentioned in the methods section and rightly pointed out, all 23 in-depth interviews were conducted qualitatively but were however, analyzed in a more quantitative manner. This was keeping in mind the funders requirement for quantifying the care seeking pathway of TB patients pre and post intervention of an interface agency. However, an in depth thematic qualitative analysis is being undertaken which will be sent on for publication shortly.

- Far more qualitative and categorical data appear to have been collected (as suggested by both supplemental files) but only a few aspects appear to be presented in this article. Similarly, there is no qualitative (or for that matter, objective) data around reasons for delay, or triangulation b/w the quant and qual findings - one would expect this in a typical mixed methods analysis. Perhaps these will be published later. If so, the authors may want to reconsider how to describe the methods and findings so it is clear at the outset what this paper specifically seeks to address. For example, they may want to highlight that although other data were collected, these data will not be assessed herein.

**Response: **As suggested, we will add the disclaimer to our Methods section which will read as, ***“Although other data was collected, it will not be assessed herein as the purpose of this publication was to focus solely on the durations and testing practices followed.”*** As you have rightly mentioned, we are currently in the process of writing a qualitative paper that highlights reasons for delays: socio demographic reasons, health seeking behavior, role of health systems, etc for DR-TB patient pathways.

- This foundational paper ^1^ should be cited and compared against when discussing the current study findings.

**Response: **Thank you for your suggestion. The foundational paper has been cited in our previous publication; (Mistry N, Rangan S, Dholakia Y, et al.: Durations and delays in care seeking, diagnosis and treatment initiation in uncomplicated Pulmonary Tuberculosis patients in Mumbai, India. PLoS One. 2016; 11(3): e0152287) wherein we have compared the pathway delays of DS-TB patients. Since pathways for DR-TB patients are much longer, our paper is focused on highlighting the increased pathways they have in terms of being diagnosed with the correct type of TB and being initiated on the appropriate treatment. 

- Were there any significant associations or trends in the data with regards to patients' sociodemographic (especially, gender and income) or other characteristics collected as part of the standardized survey? While time delay is the primary focus of this paper, it appears, it would be helpful to comment if other factors played a role.

**Response: **There was no difference noted based on socio demographic factors. However, keeping the sample size in mind, we kept the focus only on durations and resisted from attempting to highlight the lack of associations in this paper.

- The abstract suggests a 180 day delay in DRTB diagnosis (1st episode) whereas the results indicate a median 87 day delay (data not shown). Later in the discussion, the . I would advise maintaining some consistency in the ways in which these findings are reported.

**Response:**  The figure mentioned in the abstract (median 180 days) is the total pathway duration for **only new patients (1 ^st^ episode)** whereas median of 87 days mentioned in the results is from first point of care to treatment initiation for **the entire cohort**. Median of 86 days is from first point of care to diagnosis for the entire cohort (this does not come up in the discussion but is reflected in Figure 2). 

- With a sample size of 23, the authors may want to acknowledge and address the implications of conveying statistically significant results from their small sample.

**Response:** Thank you for pointing this out. We will reflect this in our discussion section as, ***“Even though the study findings show certain significant differences in pathway durations of new and re-treatment DR-TB patients, we caution that in light of the small sample size, the observations currently be interpreted as indicative.”***

 This and other clarifications will be made in the manuscript at the appropriate locations once the comments from additional referees have been received as recommended by the journal.

 Thank you once again for your valuable feedback. We hope to have addressed all your concerns.

 Sincerely,

 Nerges Mistry

# Response

**Published:** 2018-04-26

**Authors:** Nerges Mistry

**Affiliations:** The Foundation for Medical Research, India

**1. Response**: Thank you for your comment. As mentioned in the methods section and rightly pointed out, all 23 in-depth interviews were conducted qualitatively but were however, analyzed in a more quantitative manner. This was keeping in mind the funders requirement for quantifying the care seeking pathway of TB patients pre and post intervention of an interface agency. However, an in depth thematic qualitative analysis is being undertaken which will be sent on for publication shortly.

**2. Response: **As suggested, we have added the disclaimer to our Statistical analysis section, page 5 which read as, ***“Although other data was collected, it will not be assessed herein as the purpose of this publication was to focus solely on the durations and testing practices followed.”*** As you have rightly mentioned, we are currently in the process of writing a qualitative paper that highlights reasons for delays: socio demographic reasons, health seeking behavior, role of health systems, etc for DR-TB patient pathways.

**3. Response: **Thank you for your suggestion. The foundational paper has been cited in our previous publication; (Mistry N, Rangan S, Dholakia Y, et al.: Durations and delays in care seeking, diagnosis and treatment initiation in uncomplicated Pulmonary Tuberculosis patients in Mumbai, India. PLoS One. 2016; 11(3): e0152287) wherein we have compared the pathway delays of DS-TB patients. Since pathways for DR-TB patients are much longer, our paper is focused on highlighting the increased pathways they have in terms of being diagnosed with the correct type of TB and being initiated on the appropriate treatment. 

**4. Response: **There was no difference noted based on socio-demographic factors. However, keeping the sample size in mind, we kept the focus only on durations and resisted from attempting to highlight the lack of associations in this paper.

**5. Response:**  The figure mentioned in the abstract (median 180 days) is the total pathway duration for **only new patients (1 ^st^ episode)** whereas median of 87 days mentioned in the results is from first point of care to treatment initiation for **the entire cohort**. Median of 86 days is from first point of care to diagnosis for the entire cohort (this does not come up in the discussion but is reflected in Figure 2). 

**6. Response:** Thank you for pointing this out. We have reflected this in our discussion section, paragraph1 as, ***“Even though the study findings show certain significant differences in pathway durations of new and re-treatment DR-TB patients, we caution that in light of the small sample size, the observations currently be interpreted as indicative.”***

 Thank you once again for your valuable feedback. We hope to have addressed all your concerns.

 Sincerely,

 Nerges Mistry and team
